# Muscle ultrasonography in costello syndrome: unveiling new clinical insights of a complex muscular phenotype

**DOI:** 10.1186/s13023-026-04332-3

**Published:** 2026-04-23

**Authors:** Chiara Leoni, Germana Viscogliosi, Deborah Pajalunga, Valentina Trevisan, Ludovica Mondelli, Maria Luigia Angeli, Consolato Gullì, Massimo Tatti, Lucrezia Perri, Iacopo Bellani, Nicolò Lentini, Roberta Pastorino, Eliza Kuczynska, Jacopo Gervasoni, Domenico Marco Maurizio Romeo, Marika Pane, Eugenio Maria Mercuri, Serena Cecchetti, Giovanna Carpentieri, Marco Tartaglia, Giuseppe Zampino, Elisabetta Flex

**Affiliations:** 1 Center for Rare Diseases, Department of Woman and Child Health and Public Health, Fondazione Policlinico Universitario A. Gemelli, IRCCS, Largo Gemelli 8, 00168 Rome, IT Italy; 2 Università Cattolica del Sacro Cuore, Rome, Italy; 3 Department of Oncology and Molecular Medicine, Istituto Superiore di Sanità, Rome, Italy; 4 Department of Radiology, Fondazione Policlinico Universitario A. Gemelli, IRCCS, Rome, Italy; 5 School of Pediatrics, University of Milano-Bicocca, Milan, Italy; 6 Section of Hygiene, University Department of Health Sciences and Public Health, Università Cattolica del Sacro Cuore, Rome, Italy; 7 Department of Laboratory and Infectious Diseases Sciences, Fondazione Policlinico Universitario A. Gemelli IRCCS, Rome, Italy; 8 Department of Child and Adolescent Neuropsychiatry, Università Cattolica del Sacro Cuore, Rome, Italy; 9 Nemo Clinical Center, Fondazione Policlinico Universitario A. Gemelli, IRCCS, Rome, Italy; 10 Pediatric Neurology, Università Cattolica del Sacro Cuore, Rome, Italy; 11 Core Facility, Istituto Superiore di Sanità, Rome, Italy; 12 Genetics and Rare Diseases Research Division, Ospedale Pediatrico Bambino Gesù, IRCCS, Rome, Italy

**Keywords:** Costello syndrome, RASopathies, HRAS mutation, Skeletal muscle, Muscle ultrasound, Fibroadipose infiltration, Myosteatosis, Myogenic differentiation, Lipid droplets, Rare diseases.

## Abstract

**Background:**

Costello syndrome (CS) is a rare genetic disorder within the spectrum of RASopathies, caused by activating mutations in the *HRAS* gene, leading to constitutive dysregulation of the RAS/MAPK signalling pathway. Among its multisystemic manifestations, a distinctive musculoskeletal involvement is frequently observed with reduction in muscle force, pain of musculoskeletal origin and muscular hypotrophy. Moreover, abnormal histological findings with variability in size, atrophy, and prevalence of type 2-fibers have been detected on anecdotal muscular biopsies of affected individuals.

**Methods:**

We conducted a monocentric study on 20 individuals (13 females, 7 males; median age 19 years) with molecularly confirmed CS diagnosis, recruited between December 2019 and December 2022. Muscle ultrasonography (US) was performed to study muscle architecture and detect fibroadipose infiltration (FAI), a key determinant of muscle quality and functional performance on lumbar paravertebral, quadriceps (vastus medialis, vastus lateralis, rectus femoris), and gastrocnemius muscles. FAI was graded according to the Heckmatt scale (I–IV). Nutritional and metabolic assessments were conducted including macronutrient intake (3 days diet recall) and resting energy expenditure (REE). To investigate the pathogenic contribution of HRAS dysregulation to skeletal muscle development, preclinical studies were performed in engineered mouse myoblasts expressing *HRAS* p.Gly12Ser and p.Gly13Cys variants.

**Results:**

FAI was detected in 100% of participants in at least one muscle, most commonly in lumbar paravertebral muscles (85%) and vastus lateralis (70–75%); no grade IV involution cases occurred. FAI severity showed no association with age, physical activity, nutritional/biochemical parameters, or REE, but displayed district‑specific correlations with skeletal anomalies (tight Achilles tendon and coxa valga). In vitro, myoblasts expressing HRAS mutants exhibited increased proliferation, impaired myogenic differentiation/fusion, and lipid droplet accumulation with elevated cholesteryl esters compared with controls.

**Conclusions:**

US shows potential as a non-invasive tool for assessing and monitoring muscular health in CS and related RASopathies, though further data are needed to support its use. The pattern supports HRAS‑mediated metabolic dysfunction as a primary driver of FAI, warranting multicenter longitudinal studies with standardized quantitative metrics integrated with functional testing in the view of future therapeutic trials.

**Supplementary Information:**

The online version contains supplementary material available at 10.1186/s13023-026-04332-3.

## Introduction

Costello syndrome (CS, OMIM #218040) is an ultra-rare disorder, belonging to a wide group of genetic conditions identified as RASopathies. The latter share a common pathogenesis: an aberrant activation of the RAS/MAPK pathway, a major signaling cascade regulating key cellular functions such as proliferation, differentiation, and survival [1].

CS is caused by mutations in *HRAS*, a gene coding for the homonymous GTPase, which functions as a signal hub directing several signaling pathways, including RAS/MAPK cascade [2]. The clinical phenotype encompasses characteristic facial features, postnatal growth failure, cardiac anomalies, variable intellectual disability, dermatologic manifestations, and musculoskeletal involvement [3–5].

Muscle anomalies are common across RASopathies, but CS and cardio‑facio‑cutaneous syndrome (CFCS, OMIM PS115150) display more severe involvement, with reduced muscle force and mobility and musculoskeletal pain [6–10].

Anecdotal muscle biopsies have described marked fiber size variability, atrophy, and relative type‑2 fiber predominance in CS/CFCS [11]. In transgenic models (*BRAF*^L597V^ and *HRAS*^G12V^), activation of the pathway reduces fiber size and number, while MEK inhibition shows benefit [12–14].

In the last decades, the study of the musculoskeletal system has considerably developed, thanks to the improvement of radiological techniques, leading to a more accurate definition of its anatomical features and composition [15–17]. Due to their high spatial resolution and slice thickness, ultrasonography (US), CT scans and MRI are overall considered reliable tools to study morphological features of the skeletal muscle (tissue pattern or architecture). Nevertheless, they differ in terms of sensitivity, availability and costs [15, 18–20]. Imaging techniques allow the distinction between sarcopenia, generalized muscle mass loss [21, 22], and myosteatosis, i.e. excess intramuscular fat [23]. US is a validated bedside, non‑invasive modality for monitoring of muscle health, identifying pathological changes in muscle pattern or architecture, (including edema and fibrosis) [20, 21]. Several groups have characterized US patterns in genetic neuromuscular disorders to aid differential diagnosis and to longitudinally monitor fibroadipose infiltration (FAI) [24–31].

Similarly, generalized muscle hypotrophy has been evidenced across RASopathies and it is typically more severe in CS. Nevertheless, US characterization of muscle involvement in CS (and other non‑NF1 RASopathies) and its evolution over time is not routinely performed.

Our workgroup published in 2021 a paper about clinical characterization of the musculoskeletal phenotype in CS and CFC. Herein, we report the natural continuation of the previous work, moving from clinical characterization toward a functional investigation of the underlying muscular abnormalities in CS.

Through an integrated clinical and experimental approach, based on engineered mouse myoblasts expressing CS-associated variants, we provided novel insights into the pathophysiology of HRAS-related muscle involvement. We also highlighted the role of muscle ultrasonography as a promising non-invasive biomarker for patient monitoring and future therapeutic trials.

## Methods

### Clinical study

All participants with a molecularly confirmed diagnosis of CS have been recruited at the Center for Rare Diseases and Birth Defects of Policlinico Universitario Agostino Gemelli, IRCCS in Rome from December 2019 to December 2022.

The study was approved by the local Ethic Committee (ID 2462 Prot. N. 0011843/19, and ID 3717 Prot. N. 0050388/20) and signed informed consent was obtained by every patient/caregiver.

During routine follow‑up, participants underwent bilateral qualitative US evaluation of lower‑limb and paravertebral muscles. A semiquantitative visual approach was preferred over quantitative US techniques, as the latter currently lack full standardization across US systems and age groups, particularly in pediatric populations. Examinations were performed on a Samsung RS85 Prestige using a 7–11 MHz linear probe. All US examinations were performed by two radiologists with expertise in musculoskeletal imaging and muscle ultrasound, with approximately 20 and 5 years of experience, respectively. The following muscles were assessed: cervical, dorsal, and lumbar paravertebral; rectus femoris, vastus medialis, vastus lateralis; and the medial and lateral heads of the gastrocnemius. The choice of muscle groups was guided by functional relevance, feasibility, and reproducibility. Quadriceps and gastrocnemius muscles were selected as major antigravity and locomotor muscles, commonly involved in gait impairment and muscle weakness, and widely assessed in neuromuscular ultrasound protocols. Paravertebral muscles were included because of their role in postural control and the high prevalence of spinal involvement in Costello syndrome. Other muscle groups were not systematically examined due to lower reproducibility, deeper anatomical location, and reduced feasibility in a pediatric and cognitively heterogeneous population.

Long muscles were scanned at proximal, mid‑belly, and distal axial levels; spinal muscles were assessed in longitudinal and axial planes [31–33]. Participants were examined in both supine and prone positions, with extended limbs and muscles relaxed.

To reduce operator and device dependency, echogenicity and FAI were graded a priori using the Heckmatt scale (I–IV) [29, 34]: I, normal muscle; II, increased muscle echo intensity with distinct bone echo; III, clearly increased muscle echo intensity with reduced bone echo; IV, severely increased muscle echogenicity with complete loss of bone echo. In this study, grade II corresponded to a visually estimated FAI < 30% of the assessed muscle, grade III to 30–60%, and grade IV to > 60%.

Physical activity/physiotherapy at the time of the US was recorded as hours/week. A 72‑hour diet recall captured daily energy intake (KKcal) and macronutrient distribution (carbohydrates, lipids, proteins) according to LARN 2014 reference ranges [35]. Indirect calorimetry (QUARK RMR, Cosmed, Pomezia, Italy) [30] was performed in participants > 15 kg to measure REE, which was compared with theoretical basal metabolism (Schofield formula) [36]. Fasting biochemical tests (glucose, lipid and thyroid profiles, iron studies, and creatine phosphokinase -CPK-) were obtained using standard reference ranges.

### Functional studies

#### Clones, infection and differentiation

The mutations resulting in the Gly12Ser (G12S) and Gly13Cys (G13C) changes were introduced by site-directed mutagenesis in an Xpress-tagged human *HRAS* cDNA cloned in pc.DNA6-Xpress (Invitrogen). Subsequently, each of the two Costello associated mutations and WT *HRAS* were subcloned in the bicistronic retroviral vector pMX-IRES-GFP fused with Xpress tag. Constructs were transfected into Phoenix packaging cells to produce retroviruses to infect muscle satellite cells (MSC).

MSC were seeded onto gelatin-coated dishes and cultured in growth medium (Ham’s F10 Nutrient Mix, supplemented with 20% fetal bovine serum, 3% home-prepared chicken embryonic extract, 2.5 ng/ml basic-FGF, and 1% penicillin/streptomycin), at 38 °C and 7% CO2. Cells were detached from culture plates by incubation with PBS, supplemented with 0.5 mM.

H-Ras mutants were stably expressed in MSC by retroviral infections. After 96 h from infection, transgenic MSC were subjected to fluorescent activated cell sorting (FACS) according to GFP expression and highly purified cell populations were selected and expanded. The expression levels of H-Ras mutants were verified by western blot analyses using a mouse monoclonal X-press antibody (Invitrogen). Muscle differentiation was achieved by plating transgenic MSC onto gelatin-coated dishes in differentiation medium (high glucose DMEM, supplemented with 10% fetal bovine serum and 1% penicillin/streptomycin) for 72 h, at 38 °C and 7% CO2.

#### Immunofluorescence

Transgenic MSC were fixed in 4% paraformaldehyde and permeabilized with 0.25% Triton X‑100. For BrdU staining, DNA was denatured with 2 M HCl for 10 min at room temperature (RT). Primary mouse monoclonal antibodies targeted BrdU (clone Bu20a, DAKO) and myosin heavy chain (MHC) [35]. Alexa Fluor 488‑ or 594‑conjugated secondary antibodies were used; nuclei were counterstained with DAPI (0.2 µg/mL, 20 min, RT). The differentiation index was defined as nuclei in MHC‑positive cells/total nuclei; the fusion index as MHC‑positive cells with ≥ 3 nuclei/total MHC‑positive cells. Lipid droplets were stained with Bodipy after fixation (3% PFA) and permeabilization (0.5% Triton X‑100). Imaging used a Zeiss LSM980 confocal system and an Axioskop2 plus microscope.

#### Western blot analysis

Total cell lysates were prepared in RIPA buffer (500 mM NaCl). Proteins were resolved on 4–12% polyacrylamide gels and transferred for immunoblotting with anti‑Xpress (mouse monoclonal; Invitrogen), anti‑pH3S10 (rabbit polyclonal; Santa Cruz), and anti‑MHC (mouse monoclonal) [37]. β‑tubulin (mouse monoclonal; Sigma) served as loading control.

#### Lipid extraction and analysis

For each line, cells cultured in 150 cm^2^ dishes were harvested using trypsin after reaching confluency, rinsed with PBS and pelleted by centrifugation. Cell pellet was resuspended in 1 ml 0.9% NaCl and 6 ml of chloroform/methanol (2:1,v/v) were added. Mixture was vortexed vigorously and centrifuged at 1500 g for15 min, aqueous phase was discarded and organic phase was dried under N2 gas. Lipid extracts from 1.5 × 10^6^ cells were applied on HPTLC silica gel 60 plates (Merck, Darmstadt, Germany). Neutral lipids were resolved using a solvent system of hexane/diethyl ether/acetic acid (70:30:1, v/v/v) and were detected by staining with an aqueous solution containing 3% cupric acetate and 8% phosphoric acid and subsequent charring at 140 °C for 10 min. Lipids were identified by co-migration of commercially available standards and quantified using Alphaview software (Protein Simple, San Jose, CA, USA).

### Statistical analysis

Analyses were performed in R (v4.4.0, 2024‑04‑24). Categorical variables are reported as absolute frequencies and percentages, whereas quantitative variables are reported as median and interquartile range (Q1, Q3). The associations between ordinal FAI grades across muscle district and age, dietary/physical measures were assessed using Kendall’s rank correlation coefficient (τ). No multiple testing correction was applied due to the exploratory nature of the analysis. All statistical tests were two‑sided and *p* < 0.05 was considered statistically significant. Functional‑study statistics followed figure legends and used Student’s t‑test or GraphPad Prism 5; significance was denoted as **p < 0.01 and ***p < 0.001.

## Results

### Clinical study

Twenty individuals (13 females; 7 males) with CS were enrolled. Median age was 19 years (range: 10 months-38 years). The most common CS-causative *HRAS* variant p.Gly12Ser was observed in 16 (80%) individuals, the other reported ones being p.Gln22Lys (5%), p.Gly12Ala (5%), and p.Gly13Cys (10%).

Skeletal anomalies such as scoliosis and tight Achilles tendons were both detected in 14 individuals (70%) of the study population, while coxa valga subluxans was observed in 9 (45%) (Table 1).

**Table 1:** Demographic data and baseline muscle-skeletal phenotype in the study sample

Patient ID	Age	Sex	HRAS variants	PA h/week	Scoliosis (0002650)	Tight Achilles tendons (0001771)	Coxa valga (0002673)
1	10 m	F	p.Gly12Ser	0	n	y	n
2	2	M	p.Gln22Lys	0	n	n	n
3	6	F	p.Gly12Ser	1 to 2	n	y	y
4	8	F	p.Gly12Ser	1 to 2	y	y	y
5	9	F	p.Gly12Ser	1 to 2	y	y	n
6	10	M	p.Gly12Ala	1 to 2	n	y	y
7	13	F	p.Gly12Ser	> 2	y	y	y
8	15	M	p.Gly13Cys	1 to 2	y	n	n
9	17	M	p.Gly12Ser	1 to 2	y	y	n
10	19	M	p.Gly12Ser	0	n	y	y
11	19	F	p.Gly13Cys	0	y	n	n
12	20	F	p.Gly12Ser	0	y	n	y
13	22	M	p.Gly12Ser	0	y	y	n
14	23	F	p.Gly12Ser	0	y	n	n
15	23	F	p.Gly12Ser	0	y	n	n
16	26	F	p.Gly12Ser	0	y	y	y
17	28	F	p.Gly12Ser	0	y	y	n
18	33	M	p.Gly12Ser	> 2	n	y	y
19	36	F	p.Gly12Ser	0	y	y	y
20	38	F	p.Gly12Ser	0	y	y	n

ID: patient identity; PA: physical activity evaluated in number of hours per week; numbers under muscle-skeletal anomalies refer to HPO code

At evaluation, 12 (60%) patients reported no regular physical activity/physiokinesitherapy; 6 (30%) performed 1–2 h/week; and 2 (10%) performed > 2 h/week (Table 1).

### Ultrasound findings

US revealed FAI in at least one district in all participants (100%). No grade IV FAI was observed.

The youngest subject (11 months) showed grade II FAI limited to the lumbar paravertebral muscles; all other districts were unaffected. Anyway, no significant differences in muscle echogenicity were detected between the pediatric and adult sub-cohorts.

### Quadriceps femori findings

**Fig. 1:**
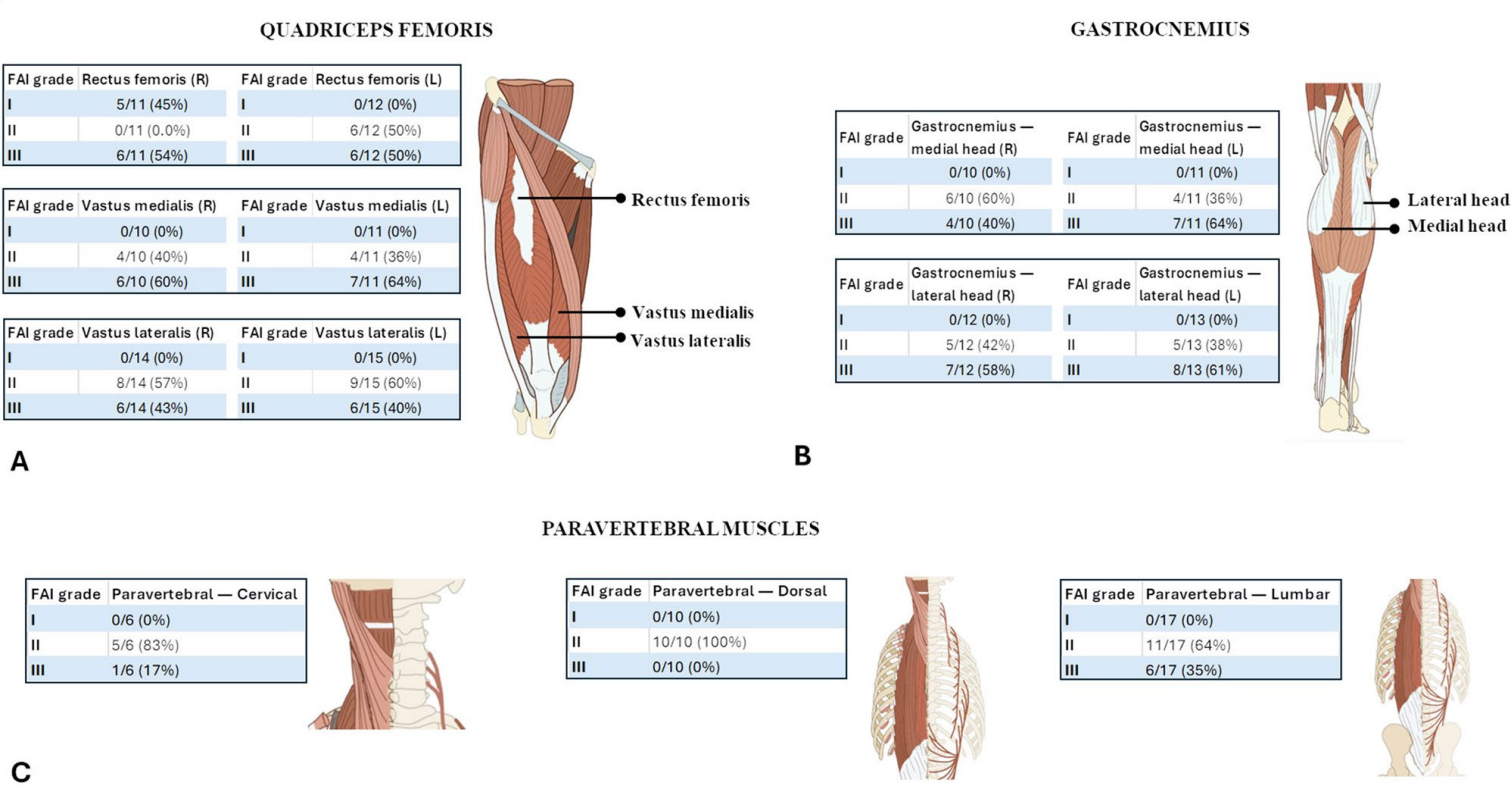
Prevalence of fibroadipose infiltration grades across muscle districts according to Heckmatt score in the cohort. Tables report the prevalence of FAI according to the Heckmatt score detected in individuals with a positive ultrasound examination, stratified by muscle district (rectus femoris, vastus medialis, vastus lateralis, medial and lateral heads of the gastrocnemius, cervical, thoracic, and lumbar paravertebral muscles) and side (left and right). The arrows indicate the topographic localization of the analyzed muscles

**Fig. 2:**
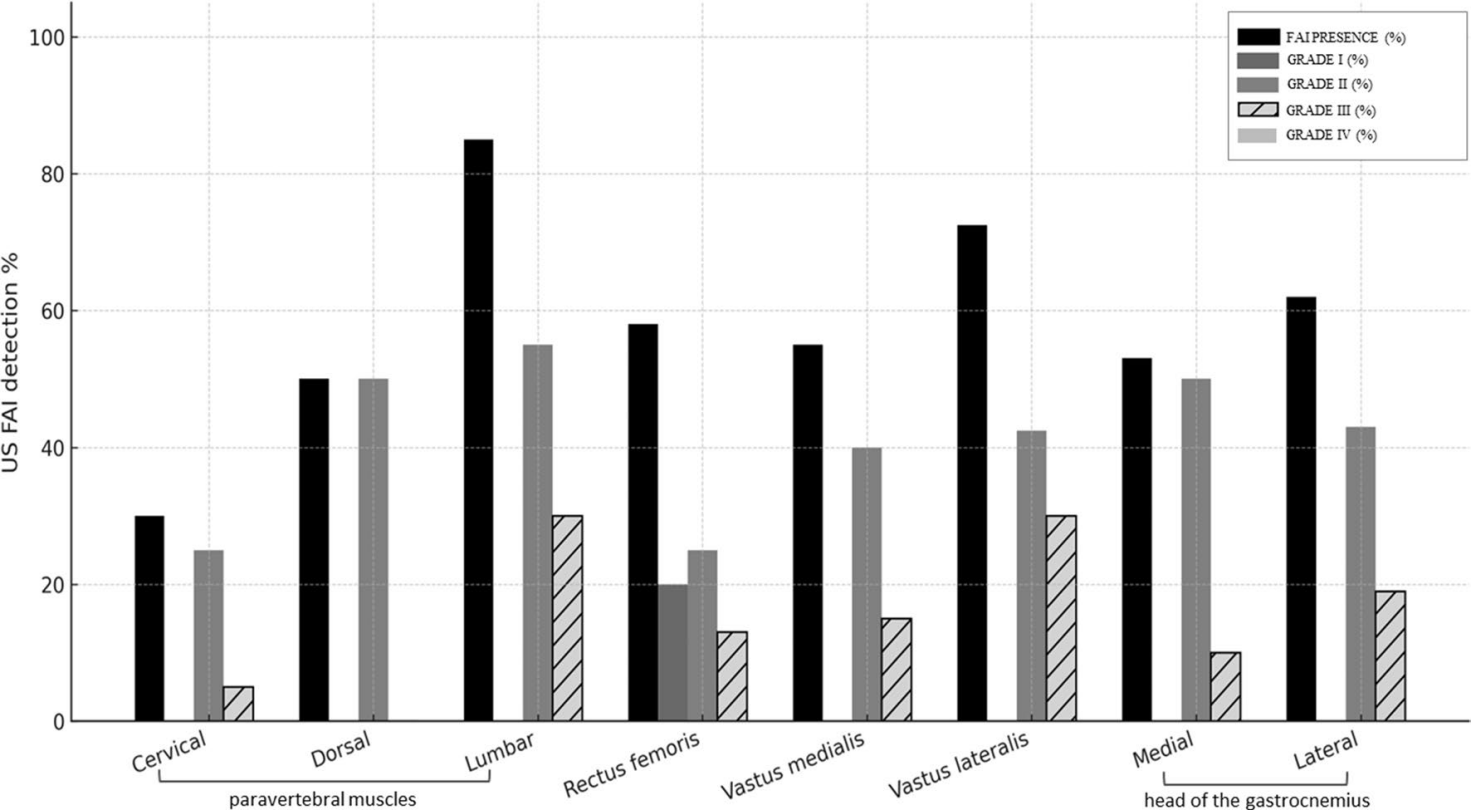
Average Fibroadipose infiltration distribution (%) across muscle groups. The figure shows the mean percentage of FAI in different muscle districts within the study cohort. For each muscle group, both the overall prevalence of FAI and the relative proportion of each severity grade (I–IV, according to the Heckmatt score) are reported. Each column represents the mean percentage calculated by averaging FAI values across the right and left sides and the proximal, medial, and distal portions of each examined muscle group

**Fig. 3:**
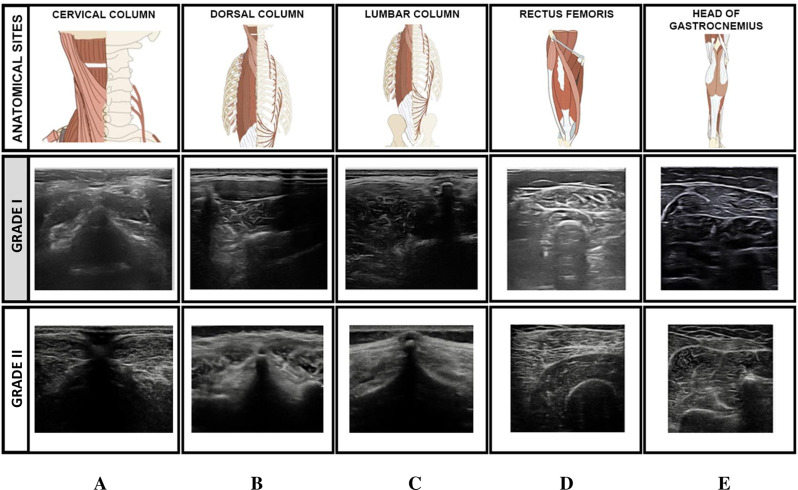
Muscle Ultrasound Images from our study cohort: comparison between Fibroadipose infiltration Grade I and II. The figure displays Ultrasound scans of the cervical, dorsal, and lumbar paravertebral muscles, the rectus femoris, and the head of the gastrocnemius. For each muscle group, Fibroadipose infiltration Grade I (normal muscle echogenicity with clear visualization of bone echo) and Fibroadipose infiltration Grade II (increased muscle echo intensity with still distinguishable bone echo, corresponding to < 30% visual fibroadipose involution) are shown. Anatomical illustrations above each column provide topographic reference for the assessed muscle districts

FAI in rectus femoris muscle was detected in about half (50%) of the studied population with a FAI grade ranging between I to III (Fig. 1A) (Fig. 2) (Fig. 3D).

Similarly, when evaluating the right vastus medialis FAI was reported in about half (50%) of the study sample ranging between FAI grade II to III (Fig. 1A) (Fig. 2).

FAI grade (mostly II to III) was detected in half (50%) of the study sample in vastus lateralis muscle (Fig. 1A) (Fig. 2).

The lateral and medial heads of the gastrocnemius were also involved bilaterally by FAI in 50% of cases, with Heckmatt score ranging between a grade II and III. (Fig. 1B) (Fig. 2) (Fig. 3E).

### Paravertebral muscles findings

Concerning paravertebral muscles, the lumbar ones were more frequently involved by FAI (85%) compared to the cervical ones (30%). In particular, cervical muscles were affected in 6 (30%) patients, (5 grade II; 1 grade III). (Fig. 1C) (Fig. 2) (Fig. 3A). Dorsal paravertebral muscles were affected in 10 (50%) patients, all grade II (Fig. 1C) (Fig. 2) (Fig. 3B). Lumbar paravertebral muscles were affected in 17 (85%) patients: 11 grade II and 6 grade III. (Fig. 1C) (Fig. 2) (Fig. 3C).

### Biochemical analysis

Fasting biochemical analysis were performed in all patients at the time of the muscular US evaluation. (Supplementary Table 1) Glycemia was below normal range (r.r. 65–100 mg/dL) in 8 (40%) patients and 6 (30%) had values at the lower limits of the range (< 70 mg/dL). Total cholesterol (r.r. 130–200 mg/dL) was slightly elevated in 3 (15%) subjects and reduced in 2 (10%). HDL (r.r. > 40 mg/dL) was below range in 1 (5%), whereas LDL (r.r. <130 mg/dL) was above range in 3 (15%) and triglycerides were above range in 1 (5%) (r.r. 20–170 mg/dL). Insulin-like growth factor-1 (IGF-1) was below range in 9 of 17 subjects (53%) (in three patients such value was not available). CPK were in range in all assessed individuals. (Supplementary Table 1).

### Metabolic data

REE was assessed in 18 (90%) individuals (#ID1 and #ID2 were excluded due to weight below the required threshold). And an elevated REE was observed in 9 (50%).

An appropriate daily Kcal intake was observed in 17 (85%) patients whereas 3 (15%) showed an increased intake. Daily carbohydrates’ intake was appropriate in 4 (20%) subjects, whereas a suboptimal intake was observed in 13 (65%), and an increased one in 3 (15%). Lipid intake was in range in 17 (85%) patients, with only 1 (5%) showing a daily intake below normal range, and 2 (10%) above the range. Finally, protein daily intake above the range was reported in 17 (85%) individuals, with the remaining subjects having normal values (Supplementary Table 2).

### Correlation analysis

No clinically relevant correlations were observed between biochemical parameters, REE, Daily Kcal intake and FAI. Significant positive correlations were observed between FAI in the quadriceps femoris and the presence of tight Achilles tendons (medial rectus femoris and proximal/medial sites of vastus lateralis, *p* < 0.001, τ = 0.76–0.86), coxa valga (medial vastus medialis and proximal/medial sites of vastus lateralis, *p* < 0.006, τ = 0.66–0.67), and age (Right Rectus Femoris distal; Right Vastus Lateralis proximal, medial, and distal; Left Rectus Femoris distal; Left Vastus Lateralis proximal, medial, and distal, *p* < 0.011, τ = 0.49–0.75).

In the gastrocnemius, FAI exhibited no significant correlations with age, the presence of tight Achilles tendons, coxa valga, scoliosis or other postural abnormalities. Furthermore, concerning the dorsal paravertebral muscles (proximal, medial and distal sites), FAI showed a significant positive correlation with the presence of coxa valga (*p* < 0.001, τ = 0.81) and age (*p* = 0.002, τ = 0.60).

### Preclinical study

#### HRAS mutants hinder muscle differentiation in vitro

**Fig. 4:**
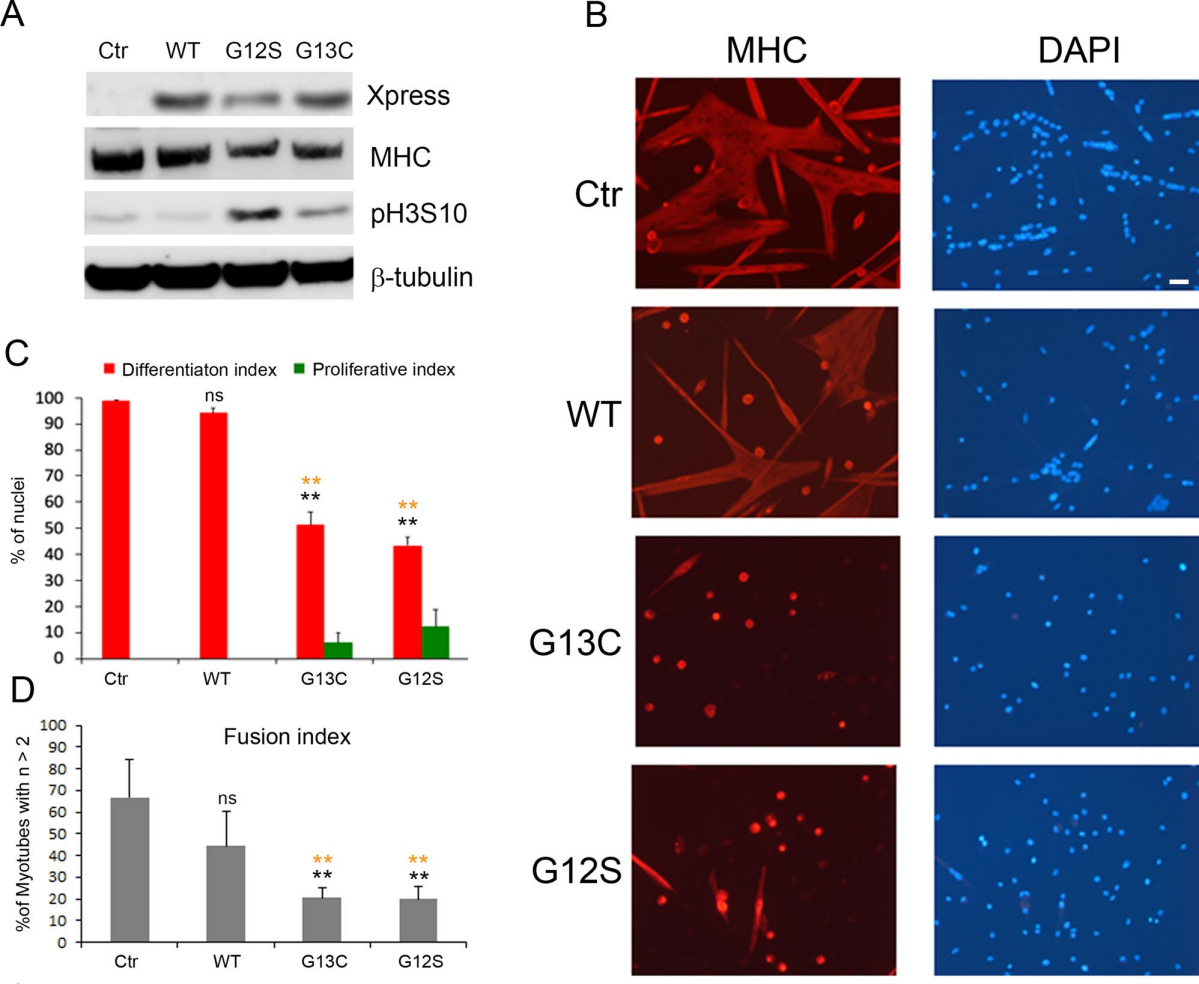
Differentiation assay of mouse myoblasts stable expressing HRAS WT and mutants **A)** Representative western blot analyses show expression levels of Xpress-tagged HRAS WT and mutant proteins in mouse myoblasts. pH3S10 is shown as a proliferation marker and myosin heavy chain (MHC) as a late differentiation marker. Membranes were immunoblotted with a mouse monoclonal anti-Xpress antibody, a rabbit polyclonal anti-pH3S10 antibody and a mouse monoclonal anti-MHC antibody. Mouse monoclonal anti-β-tubulin antibody was used for protein normalization. **(B)** Immunofluorescence to MHC and DAPI staining show that myoblasts expressing HRAS mutants fail to start the differentiation process. Myoblasts able to bypass this differentiation block become mononucleated myocytes, express low levels of MHC, and fail to fuse into multinucleated myotubes. **(C)** Graph quantifying differentiation (red) and cell cycle exit (green) efficiency (differentiation index = n. of nuclei in MHC positive cells /n. of nuclei in total cells; proliferative index = n. of BrdU positive nuclei /n. of total nuclei in MHC negative cells). 5-bromo-2’-deoxyuridine, BrdU, is a synthetic nucleoside analogue with a chemical structure similar to thymidine and is incorporated in nascent DNA strands during DNA replication. Average ± SD of three biological replicas (∗∗*p* < 0.01, Student t-test). **(D)** Graph quantifying fusion capability (fusion index = n. of MHC+ cells with nuclei > 2/n. total MHC+ cells). Average ± SD of three biological replicas (∗∗*p* < 0.01, Student t-test). Black asterisks indicate statistically significant differences between HRAS expressing cells and Ctr cells; orange asterisks indicate statistically significant differences between cells overexpressing HRAS mutants and cell expressing HRAS wild-type; ns = not significant

Costello syndrome is caused by heterozygous germline gain-of-function mutations in the GTPase HRAS, which increase active GTP-bound levels while total protein expression remains overall unaltered. Therefore, to evaluate the direct involvement of *HRAS* in skeletal muscle defects, we engineered, through retrovirus infection, a mouse myoblast cell line to stably express either WT or two recurrent *HRAS* mutations (p.Gly12Ser and p.Gly13Cys) identified in CS patients. Initially, fluorescence-activated cell sorting was performed to isolate highly purified myoblast populations. Subsequently, although in this model HRAS protein levels do not reflect the physiological protein content, we verified cells populations overexpressing comparable levels of WT and p.Gly12Ser and p.Gly13Cys HRAS mutants, by western blot analysis (Fig. 4A). In order to characterize the role of such HRAS mutants in muscle differentiation, we measured the expression of a late differentiation myogenic marker, myosin heavy chain (MHC), 72 h after switching the engineered myoblast cell lines from proliferative to differentiative medium conditions. MHC protein levels were reduced following HRAS mutants expression in comparison to the WT proto-oncogene endowed cells and control ones, suggesting an impairment of muscle differentiation process (Fig. 4A). In addition, the levels of pH3S10, a proliferative marker [38] were increased following the expression of both HRAS mutants, indicating a deregulation of the cell cycle control (Fig. 4A). To better characterize the effect of p.Gly12S and p.Gly13Cys HRAS mutants in muscle differentiation, immunofluorescence technique was used to morphologically evaluate muscle differentiation parameters. Figure 4B and C show that several mouse myoblasts stably expressing HRAS mutants failed to express MHC and exit the cell cycle when subjected to differentiation condition. Moreover, myoblasts able to bypass this differentiation block appeared as mononucleated MHC positive myocytes due to a reduced fusion potential (Fig. 4B, C, and D). Interestingly, the differentiation capability of myoblasts expressing the WT form of HRAS was unchanged in comparison to control cells (Fig. 4B, C, and D).

### Increased lipid droplets in p.Gly12S and p.Gly13Cys HRAS engineered myoblasts

**Fig. 5:**
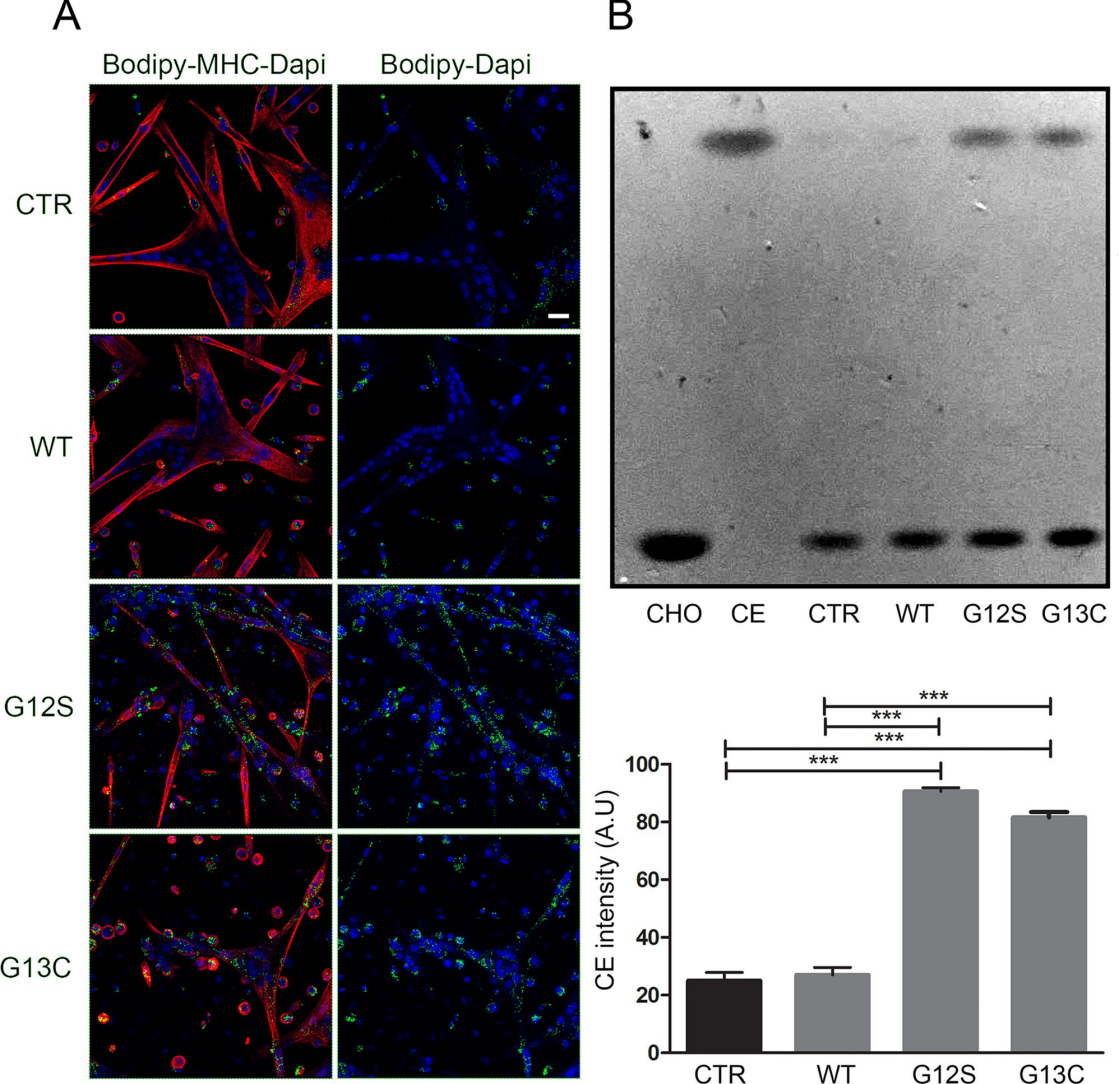
Mouse myoblasts stable expressing HRAS mutants showed an increase of fatty acid synthesis and intracellular storage as lipid droplets. Mouse myoblasts stable expressing HRAS mutants showed an increase of fatty acid synthesis and intracellular storage as lipid droplets. (**A**) Confocal laser scanning microscopy analysis shows an increased amount of lipid droplets in mouse myoblasts expressing HRAS mutants (G12S and G13C) compared to cells expressing HRAS WT or control cells. Lipid droplets content was evaluated after fixation with 3% PFA and permeabilization with 0.5% Triton X-100 by using Bodipy dye (green). Cells were stained also with myosin heavy chain (MHC) antibody (red) to confirm the differentiation defect promoted by HRAS mutants expression. Nuclei are visualized by DAPI staining (blue). Scale bar is 20 μm. (**B**) High-performance thin-layer chromatography shows variable higher cholesterol esters (CE) levels in mouse myoblasts stable expressing HRAS mutants compared to WT or control cells. The relative CE intensities were expressed as arbitrary units (a.u.) normalized to the cell number. The corresponding graphs show the mean ± SE of three independent experiments. p value (∗∗∗*P* < 0.001) were calculated by one-way ANOVA with Tukey’s correction for multiple testing

Based on our prior observation of increased lipid droplets in patient fibroblasts [39] and reports of lipid accumulation in undifferentiated muscle cells in altered myogenesis models [40], we evaluated the presence of lipid bodies in HRAS expressing muscle cells in both proliferating and differentiated conditions. Using the fluorescent lipid-specific Bodipy dye, we found that all proliferating myoblast cell lines exhibited cytoplasmic lipid droplets (Fig. S1). Such evidence is in accordance with previous findings showing that lipid droplets are not detected in quiescent muscle satellite cells, but arise upon their activation and proliferation [41]. Interestingly, Bodipy staining revealed larger and more abundant lipid droplets in the cytoplasms of p.Gly12S and p.Gly13Cys HRAS expressing cells in comparison to WT HRAS endowed or control ones (Fig. S1). In order to investigate lipid bodies levels in differentiated cells, we induced proliferating myoblasts to differentiate. Using a confocal laser scan microscopy, we showed a significant increased amount of lipid droplets in both p.Gly12S and p.Gly13Cys HRAS expressing cultures compared to WT and control ones (Fig. 5A). High-performance thin-layer chromatography performed with parallel series of engineered cell cultures confirmed such lipid accumulation showing a variable higher cholesterol esters (CE) levels in cells expressing HRAS mutants compared to WT HRAS endowed and control ones (Fig. 5B).

## Discussion

The RAS/Mitogen activated protein kinase (MAPK) pathway represents a complex intracellular network, consisting of several effectors and regulators, which control cell growth, division, differentiation, and senescence [1]. Autosomal dominant or recessive variants in gene coding for regulators and/or effectors belonging to this pathway, lead to RASopathies,- a wide group of rare disorders, which might have unique features or overlapping symptoms, sharing an altered multisystemic development, affecting also skeletal muscles [42] .

Generalized hypotonia is one of the most frequent clinical features reported in RASopathies from birth, with the highest prevalence in CS and CFCS [6]. Most patients require early and prolonged physical therapy to improve muscle tone and to acquire neurological milestones. Pathophysiological basis of hypotonia in RASopathies remains debated, with some authors speculating that hypotonia in RASopathies might be related to myopathy, others to abnormal muscle fiber size detected in muscular biopsies [10–12].

Muscle hypotrophy and physical weakness have also been reported with a decreased muscle force (evaluated by dynamometer and vigorimeter) in RASopathies, associated with co-factors such as neuromotor and cognitive delay and poor patient’s compliance [3, 4, 6, 8, 10].

The musculoskeletal characteristics in CS were largely outlined by our workgroup and other researchers in literature, with findings showing generalized muscle hypotrophy and skeletal malformations [3–5, 8]. Previous studies on DXA scan in CS individuals documented decreased bone mineral density and a significantly reduced lean mass compared to age and sex-matched controls, but no differences were observed concerning fat mass [43, 44].

More recently, it has been showed that also individuals with Noonan syndrome (NS) had lower lean mass compared to reference values, with progressive muscle mass decline along life [45].

In this context, this study provides integrated clinical and preclinical evidences for HRAS‑driven muscle involvement in CS. FAI was detected in all individuals through US, with predominant involvement of lumbar paravertebral and vastus lateralis muscles; interestingly, grade IV involution was never observed.

FAI severity did not associate with age, physical activity, nutritional or biochemical indices, or REE, but exhibited some district‑specific correlations with skeletal anomalies (tight Achilles tendon, coxa valga). We speculate low IGF-1 levels might further contribute to muscle hypotrophy in CS by reducing anabolic signaling and satellite cell activation, thereby amplifying the effects of HRAS-driven metabolic dysregulation on muscle structure. Together with normal CPK levels, frequent hypoglycemia and altered lipid profiles, these findings support a predominantly metabolic rather than purely degenerative mechanism of muscle involvement. Our preclinical data align with this interpretation. HRAS‑mutant myoblasts showed maintenance of proliferative capacity, impaired differentiation and fusion, and lipid droplet accumulation with elevated cholesteryl esters. Prior studies in RASopathy models have demonstrated that identified variants in *HRAS*,* BRAF*,* MAP2K1* and *NF1* gene might promote an aberrant myoblast differentiation, reducing the numbers of muscle fibers, myotube and level of myosin heavy chain protein [10, 46]. Moreover, NF1 mouse model showed an increased lipids deposits in muscle biopsies. However, this finding was not reported in CS and CFCS models yet. Recent studies on animal models confirmed that the RAS/MAPK signaling pathway is involved in skeletal muscle development. Pathway activation has a dual effect: to stimulate proliferation of muscle precursor cells and, suppress at the same time, myoblasts differentiation [12–14].

In this framework, Carpentieri et al. recently speculated that the combination of hypoglycemia, hypercholesterolemia and increased REE, three key features in CS, might be the consequence of an increased activity of the glucose transporter (GLUT4) caused by a ROS-dependent AMPKa and p38 activation [39]. Authors prompted the hypothesis that hyperactivation of GLUT4, resulting in an increased glucose uptake into cells, led to enhanced glycolysis, with increase in fatty acids production and their storage as cytoplasmic lipid droplets in patient’ fibroblasts. In agreement, we found that proliferating myoblasts engineered to express HRAS mutants showed larger and more abundant lipid droplets in their cytoplasm, suggesting that the lipid metabolic defects arise at this undifferentiated state. This observation led us to hypothesize that cytoplasmic lipid droplets accumulation observed in differentiated cells expressing HRAS mutants does not result from an impaired muscle differentiation but derives from a metabolic imbalance that may contribute to cell cycle control deregulation and differentiation defects.

Based on such pre-clinical data, we hypothesize that the fibroadipose infiltration (FAI) visualized in muscle tissues of CS patients might be generated, at least in part, by the anomalous cytoplasmic fat storage of patients’ muscle cells. This aberrant fat accumulation, affecting tissue health and function, has been observed in other conditions such as aging, metabolic disease and insulin resistance [47, 48]. Unfortunately, the US technique allowed us to appreciate only the amount and body localization of the aberrant fat storage, but it does not enable discrimination between intracellular lipid deposits and fat localized within the connective tissue surrounding muscle fibers.

In the general population, fibroadipose infiltration and sarcopenia, both hallmarks of aging muscle, are associated with altered ultrasonographic parameters, particularly in lower limb muscles [17, 22]. Notably, in our cohort, FAI of variable degree was already evident in pediatric patients, including very young individuals, strongly supporting the hypothesis that HRAS upregulation is a primary driver of premature muscle FAI in CS rather than a secondary age-related phenomenon. Considered the high prevalence of FAI in our cohort, a repeatable, widely available, non‑irradiating methodology as US might be necessary for risk stratification and longitudinal monitoring in CS and related RASopathies.

The detection of FAI even in very young patients, alongside the complete absence of grade IV changes, underscores that ultrasonography can identify muscle abnormalities at an early, potentially reversible stage. This supports its role not only as a clinical monitoring tool but also as a sensitive and clinically meaningful endpoint in future interventional trials. Although quantitative ultrasound techniques such as grayscale analysis or elastography may provide objective metrics, their use is still limited by system dependency and lack of standardized reference values. Future multicenter studies should integrate quantitative ultrasound with functional measures (e.g., 6‑minute walk test, handgrip strength) to further strengthen its role as an imaging biomarker, and to anchor imaging changes to patient‑centered outcomes.

Limitations of the study include the qualitative nature of US grading, which may introduce subjectivity in assessment; inherent operator and system dependency, which could affect measurement reproducibility; and the small sample size, reflecting the rarity of the disease, which may limit statistical power and the generalizability of the findings. Another major limitation of this study is its cross-sectional design. No longitudinal muscle ultrasound data were available, precluding the assessment of temporal changes in fibroadipose infiltration during the clinical course. Together, these factors may reduce precision and obscure modest associations with potentially modifiable factors such as age, diet, or physical activity. To overcome these limitations, future multicenter longitudinal studies with harmonized imaging protocols, systematic inter-rater reliability assessments, and prespecified quantitative endpoints are warranted, which would allow more robust evaluation of clinical and functional associations.

## Conclusions

Muscle US identified FAI in all individuals with Costello syndrome, predominantly affecting lumbar paravertebral muscles and vastus lateralis, with no grade IV involvement. FAI severity was not associated with age, physical activity, nutritional/biochemical measures, or REE, but showed site‑specific correlations with selected skeletal anomalies (tight Achilles tendon and coxa valga). In myoblast models expressing HRAS p.Gly12Ser or p.Gly13Cys, we observed increased proliferation, impaired myogenic differentiation and fusion, and marked lipid droplet accumulation with elevated cholesteryl esters, consistent with HRAS-driven metabolic dysregulation. Collectively, these data suggest that in the next future US might represent a rapid, non-invasive, and repeatable approach for stratification, longitudinal monitoring, and endpoint selection in studies of CS and related RASopathies. The pattern suggests FAI is primarily linked to HRAS-mediated metabolic dysfunction rather than modifiable factors, highlighting the need for multicenter longitudinal studies with standardized quantitative metrics integrated with functional testing.

## Supplementary Information

Below is the link to the electronic supplementary material.

**Supplementary Material 1:** Fasting biochemical parameters at the time of ultrasonography evaluationThe table summarizes the distribution of key biochemical markers in the study cohort, including lower and higher recorded values, median, and reference ranges. Parameters assessed included fasting glycemia, triglycerides (TAGs), total cholesterol, HDL, LDL, and insulin-like growth factor-1 (IGF-1). Reference values are age-adjusted according to BC Children’s Hospital guidelines (https://www.bcchildrens.ca/endocrinology-diabetes-site/documents/igf1nml.pdf)

**Supplementary Material 2:** Daily macronutrients kilocaloric intake according to reference range The table shows the distribution of participants with appropriate, below-range, or above-range daily caloric intake for carbohydrates, lipids, and proteins

**Supplementary Material 3:** Lipid droplets detection in proliferating muscle cells stably expressing WT and mutants HRAS Representative images showing an increased amount of lipid droplets in proliferating myoblasts expressing HRAS mutants (G12S and G13C) compared to cells expressing WT HRAS and control ones. Lipid droplets content was evaluated after fixation with 3% PFA and permeabilization with 0.5% Triton X-100 by using the fluorescent lipid-specific Bodipy dye (green). Cells were stained also with myosin heavy chain (MHC) antibody (red). Nuclei are visualized by DAPI staining (blue). Scale bar is 20 μm.

## Data Availability

Data and materials are available from the corresponding author upon request.

## Abbreviations

CS: Costello Syndrome
FAI: Fibroadipose infiltration
IGF-1: Insulin-like growth factor 1 US: Ultrasonography
REE: Resting Energy Expenditure

## References

[1] Hebron KE, Hernandez ER, Yohe ME. The RASopathies: from pathogenetics to therapeutics. Dis Model Mech. 2022;15(2):dmm049107. 10.1242/dmm.049107. PubMed PMID: 35178568; PubMed Central PMCID: PMC8862741. 35178568 10.1242/dmm.049107 PMC8862741

[2] Aoki Y, Niihori T, Kawame H, Kurosawa K, Ohashi H, Tanaka Y, et al. Germline mutations in HRAS proto-oncogene cause Costello syndrome. Nat Genet. 2005;37(10):1038–40. 10.1038/ng1641. 16170316 10.1038/ng1641

[3] Leoni C, Viscogliosi G, Tartaglia M, Aoki Y, Zampino G. Multidisciplinary Management of Costello Syndrome: Current Perspectives. JMDH. 2022;15:1277–96. 10.2147/JMDH.S291757. 35677617 10.2147/JMDH.S291757 PMC9169840

[4] Leoni C, Viscogliosi G, Stevenson DA. Orthopedic issues of the RASopathies. In: Rauen KA, editor. The RASopathies [Internet]. Cham: Springer Nature Switzerland; 2024 [cited 2026 Jan 22]. pp. 795–817. Available from: https://link.springer.com/10.1007/978-3-031-62945-7_36.

[5] Gripp KW, Morse LA, Axelrad M, Chatfield KC, Chidekel A, Dobyns W, et al. Costello syndrome: Clinical phenotype, genotype, and management guidelines. Am J Med Genet Pt A. 2019;179(9):1725–44. 10.1002/ajmg.a.61270. 10.1002/ajmg.a.61270 PMC8238015 31222966

[6] Johnson B, Goldberg-Strassler D, Gripp K, Thacker M, Leoni C, Stevenson D. Function and disability in children with Costello syndrome and Cardiofaciocutaneous syndrome. Am J Med Genet Pt A. 2015;167(1):40–4. 10.1002/ajmg.a.36828. 10.1002/ajmg.a.36828 25346259

[7] Leoni C, Triumbari EKA, Vollono C, Onesimo R, Podagrosi M, Giorgio V, et al. Pain in individuals with RASopathies: Prevalence and clinical characterization in a sample of 80 affected patients. Am J Med Genet Pt A. 2019;179(6):940–7. 10.1002/ajmg.a.61111. 10.1002/ajmg.a.61111 30854769

[8] Leoni C, Romeo DM, Pelliccioni M, Di Già M, Onesimo R, Giorgio V, et al. Musculo-skeletal phenotype of Costello syndrome and cardio-facio-cutaneous syndrome: insights on the functional assessment status. Orphanet J Rare Dis. 2021;16(1):43. 10.1186/s13023-021-01674-y. 33482860 10.1186/s13023-021-01674-y PMC7821553

[9] Hopkins E, Lin AE, Krepkovich KE, Axelrad ME, Sol-Church K, Stabley DL, et al. Living with Costello syndrome: Quality of life issues in older individuals. Am J Med Genet Pt A. 2010;152A(1):84–90. 10.1002/ajmg.a.33147. 10.1002/ajmg.a.33147 20034064

[10] Stevenson DA, Allen S, Tidyman WE, Carey JC, Viskochil DH, Stevens A, et al. Peripheral muscle weakness in RASopathies. Muscle Nerve. 2012;46(3):394–9. 10.1002/mus.23324. 22907230 10.1002/mus.23324

[11] Tidyman WE, Lee HS, Rauen KA. Skeletal muscle pathology in Costello and cardio-facio‐cutaneous syndromes: Developmental consequences of germline Ras/MAPK activation on myogenesis. Am J Med Genet Pt C. 2011;157(2):104–14. 10.1002/ajmg.c.30298. 10.1002/ajmg.c.30298 21495178

[12] Maeda Y, Tidyman WE, Ander BP, Pritchard CA, Rauen KA. Ras/ MAPK dysregulation in development causes a skeletal myopathy in an activating *Braf*^*L597V*^ mouse model for cardio-facio‐cutaneous syndrome. Dev Dyn. 2021;250(8):1074–95. 10.1002/dvdy.309. 33522658 10.1002/dvdy.309

[13] Tidyman WE, Goodwin AF, Maeda Y, Klein OD, Rauen KA. MEK-inhibitor-mediated rescue of skeletal myopathy caused by activating Hras mutation in a Costello syndrome mouse model. Dis Models Mech. 2021. 10.1242/dmm.049166. dmm.049166. 10.1242/dmm.049166 PMC8617311 34553752

[14] Rauen KA, Tidyman WE. RASopathies – what they reveal about RAS/MAPK signaling in skeletal muscle development. Dis Models Mech. 2024;17(6):dmm050609. 10.1242/dmm.050609. 10.1242/dmm.050609 PMC11179721 38847227

[15] Engelke K, Museyko O, Wang L, Laredo JD. Quantitative analysis of skeletal muscle by computed tomography imaging—State of the art. J Orthop Translation. 2018;15:91–103. 10.1016/j.jot.2018.10.004. 10.1016/j.jot.2018.10.004 PMC6260391 30533385

[16] Pirri C, Pirri N, Guidolin D, Macchi V, Porzionato A, De Caro R, et al. Ultrasound Imaging of Thoracolumbar Fascia Thickness: Chronic Non-Specific Lower Back Pain versus Healthy Subjects; A Sign of a Frozen Back? Diagnostics. 2023;13(8):1436. 10.3390/diagnostics13081436. 37189537 10.3390/diagnostics13081436 PMC10137552

[17] Fischer A, Anwar M, Hertwig A, Hahn R, Pesta M, Timmermann I, et al. Ultrasound method of the USVALID study to measure subcutaneous adipose tissue and muscle thickness on the thigh and upper arm: An illustrated step-by-step guide. Clin Nutr Experimental. 2020;32:38–73. 10.1016/j.yclnex.2020.06.003.

[18] Pillen S, Arts IMP, Zwarts MJ. Muscle ultrasound in neuromuscular disorders. Muscle Nerve. 2008;37(6):679–93. 10.1002/mus.21015. 18506712 10.1002/mus.21015

[19] Pillen S, Van Alfen N. Skeletal muscle ultrasound. Neurol Res. 2011;33(10):1016–24. 10.1179/1743132811Y.0000000010. 22196753 10.1179/1743132811Y.0000000010

[20] Pillen S, Boon A, Van Alfen N. Muscle ultrasound. Handb Clin Neurol. 2016;136:843–53. 10.1016/B978-0-444-53486-6.00042-9. PubMed PMID: 27430445. 27430445 10.1016/B978-0-444-53486-6.00042-9

[21] Petric M, Jordan T, Karteek P, Licen S, Trotovsek B, Tomazic A. Radiological assessment of skeletal muscle index and myosteatosis and their impact postoperative outcomes after liver transplantation. Radiol Oncol. 2023;57(2):168–77. 10.2478/raon-2023-0025. 37341202 10.2478/raon-2023-0025 PMC10286898

[22] Sayer AA, Cruz-Jentoft A. Sarcopenia definition, diagnosis and treatment: consensus is growing. Age Ageing. 2022;51(10):afac220. 10.1093/ageing/afac220. 36273495 10.1093/ageing/afac220 PMC9588427

[23] Henin G, Loumaye A, Leclercq IA, Lanthier N, Myosteatosis. Diagnosis, pathophysiology and consequences in metabolic dysfunction-associated steatotic liver disease. JHEP Rep. 2024;6(2):100963. 10.1016/j.jhepr.2023.100963. 38322420 10.1016/j.jhepr.2023.100963 PMC10844870

[24] Habers GEA, Van Brussel M, Bhansing KJ, Hoppenreijs EP, Janssen AJWM, Van Royen-Kerkhof A, et al. Quantitative muscle ultrasonography in the follow‐up of juvenile dermatomyositis. Muscle Nerve. 2015;52(4):540–6. 10.1002/mus.24564. 25557638 10.1002/mus.24564

[25] Tan SY, Tan CY, Yahya MA, Low SC, Shahrizaila N, Goh KJ. Quantitative muscle ultrasound as a disease biomarker in hereditary transthyretin amyloidosis with polyneuropathy. Neurol Sci. 2024;45(7):3449–59. 10.1007/s10072-024-07340-y. 38270729 10.1007/s10072-024-07340-y

[26] Katzberg HD, Bril V, Breiner A. Ultrasound in Neuromuscular Disorders. J Clin Neurophysiol. 2016;33(2):80–5. 10.1097/WNP.0000000000000234. 27035247 10.1097/WNP.0000000000000234

[27] Wijntjes J, Van Der Hoeven J, Saris CGJ, Doorduin J, Van Alfen N. Visual versus quantitative analysis of muscle ultrasound in neuromuscular disease. Muscle Nerve. 2022;66(3):253–61. 10.1002/mus.27669. 35765226 10.1002/mus.27669 PMC9545111

[28] Vincenten SCC, Teeselink S, Voermans NC, Van Engelen BGM, Mul K, Van Alfen N. Establishing the role of muscle ultrasound as an imaging biomarker in facioscapulohumeral muscular dystrophy. Neuromuscul Disord. 2023;33(12):936–44. 10.1016/j.nmd.2023.10.015. 37968164 10.1016/j.nmd.2023.10.015

[29] Moreta MC, Fleet A, Reebye R, McKernan G, Berger M, Farag J, et al. Reliability and Validity of the Modified Heckmatt Scale in Evaluating Muscle Changes With Ultrasound in Spasticity. Archives Rehabilitation Res Clin Translation. 2020;2(4):100071. 10.1016/j.arrct.2020.100071. 10.1016/j.arrct.2020.100071 PMC7853393 33543098

[30] Harada R, Taniguchi-Ikeda M, Nagasaka M, Nishii T, Inui A, Yamamoto T, et al. Assessment of the upper limb muscles in patients with Fukuyama muscular dystrophy: Noninvasive assessment using visual ultrasound muscle analysis and shear wave elastography. Neuromuscul Disord. 2022;32(9):754–62. 10.1016/j.nmd.2022.05.004. 35902324 10.1016/j.nmd.2022.05.004

[31] Maurits NM, Bollen AE, Windhausen A, De Jager AEJ, Van Der Hoeven JH. Muscle ultrasound analysis: normal values and differentiation between myopathies and neuropathies. Ultrasound Med Biol. 2003;29(2):215–25. 10.1016/S0301-5629(02)00758-5. 12659909 10.1016/s0301-5629(02)00758-5

[32] Mateos-Angulo A, Galán-Mercant A, Cuesta-Vargas AI. Ultrasound Muscle Assessment and Nutritional Status in Institutionalized Older Adults: A Pilot Study. Nutrients. 2019;11(6):1247. 10.3390/nu11061247 PubMed PMID: 31159255; PubMed Central PMCID: PMC6627854. 31159255 10.3390/nu11061247 PMC6627854

[33] Scholten RR, Pillen S, Verrips A, Zwarts MJ. Quantitative ultrasonography of skeletal muscles in children: normal values. Muscle Nerve. 2003;27(6):693–8. 10.1002/mus.10384. PubMed PMID: 12766980. 12766980 10.1002/mus.10384

[34] Heckmatt JZ, Leeman S, Dubowitz V. Ultrasound imaging in the diagnosis of muscle disease. J Pediatr. 1982;101(5):656–60. 10.1016/S0022-3476(82)80286-2. 7131136 10.1016/s0022-3476(82)80286-2

[35] Società Italiana di Nutrizione Umana (SINU). LARN—Livelli di Assunzione di Riferimento di Nutrienti ed Energia per la popolazione Italiana. IV revisione. Milano: SICS; 2014.

[36] Schofield WN. Predicting basal metabolic rate, new standards and review of previous work. Hum Nutr Clin Nutr. 1985;39(Suppl 1):5–41. PubMed PMID: 4044297. 4044297

[37] Bader D, Masaki T, Fischman DA. Immunochemical analysis of myosin heavy chain during avian myogenesis in vivo and in vitro. J Cell Biol. 1982;95(3):763–70. 10.1083/jcb.95.3.763. 6185504 10.1083/jcb.95.3.763 PMC2112936

[38] Prigent C, Dimitrov S. Phosphorylation of serine 10 in histone H3, what for? J Cell Sci. 2003;116(Pt 18):3677–85. 10.1242/jcs.00735. PubMed PMID: 12917355. 12917355 10.1242/jcs.00735

[39] Carpentieri G, Leoni C, Pietraforte D, Cecchetti S, Iorio E, Belardo A, et al. Hyperactive HRAS dysregulates energetic metabolism in fibroblasts from patients with Costello syndrome via enhanced production of reactive oxidizing species. Hum Mol Genet. 2022;31(4):561–75. 10.1093/hmg/ddab. 270 PubMed PMID: 34508588. 34508588 10.1093/hmg/ddab270

[40] Al Saedi A, Debruin DA, Hayes A, Hamrick M. Lipid metabolism in sarcopenia. Bone. 2022;164:116539. 10.1016/j.bone.2022.116539. 36007811 10.1016/j.bone.2022.116539

[41] Chen J, Markworth JF, Ferreira C, Zhang C, Kuang S. Lipid droplets as cell fate determinants in skeletal muscle. Trends Endocrinol Metabolism. 2025;36(7):645–59. 10.1016/j.tem.2024.10.006. 10.1016/j.tem.2024.10.006 PMC12116854 39613547

[42] Tidyman WE, Rauen KA. The RASopathies: developmental syndromes of Ras/MAPK pathway dysregulation. Curr Opin Genet Dev. 2009;19(3):230–6. 10.1016/j.gde. 2009.04.001 PubMed PMID: 19467855; PubMed Central PMCID: PMC2743116. 19467855 10.1016/j.gde.2009.04.001 PMC2743116

[43] Leoni C, Stevenson DA, Martini L, De Sanctis R, Mascolo G, Pantaleoni F, et al. Decreased bone mineral density in Costello syndrome. Mol Genet Metab. 2014;111(1):41–5. 10.1016/j.ymgme.2013.08.007. 24246682 10.1016/j.ymgme.2013.08.007

[44] Leoni C, Bisanti C, Viscogliosi G, Onesimo R, Massese M, Giorgio V, et al. Bone tissue homeostasis and risk of fractures in Costello syndrome: A 4-year follow-up study. Am J Med Genet A. 2022;188(2):422–30. 10.1002/ajmg.a.62615. PubMed PMID: 34913244. 34913244 10.1002/ajmg.a.62615

[45] Delagrange M, Rousseau V, Cessans C, Pienkowski C, Oliver I, Jouret B, et al. Low bone mass in Noonan syndrome children correlates with decreased muscle mass and low IGF-1 levels. Bone. 2021;153:116170. 10.1016/j.bone.2021.116170. 34492361 10.1016/j.bone.2021.116170

[46] Kossler N, Stricker S, Rödelsperger C, Robinson PN, Kim J, Dietrich C, et al. Neurofibromin (Nf1) is required for skeletal muscle development. Hum Mol Genet. 2011;20(14):2697–709. 10.1093/hmg/ddr149. PubMed PMID: 21478499; PubMed Central PMCID: PMC3118757. 21478499 10.1093/hmg/ddr149 PMC3118757

[47] Borén J, Taskinen MR, Olofsson SO, Levin M. Ectopic lipid storage and insulin resistance: a harmful relationship. J Intern Med. 2013;274(1):25–40. 10.1111/joim.12071. PubMed PMID: 23551521. 23551521 10.1111/joim.12071

[48] Jang SY, Choi KM. Impact of Adipose Tissue and Lipids on Skeletal Muscle in Sarcopenia. J Cachexia Sarcopenia Muscle. 2025;16(4):e70000. 10.1002/jcsm.70000. PubMed PMID: 40641114; PubMed Central PMCID: PMC12246390. 40641114 10.1002/jcsm.70000 PMC12246390

