# Supplementary material for: Muscle ultrasonography in costello syndrome: unveiling new clinical insights of a complex muscular phenotype
Source: Orphanet J Rare Dis. 2026 Apr 23;21:223. doi: 10.1186/s13023-026-04332-3 (PMC13277121; doi:10.1186/s13023-026-04332-3)
Supplement: Supplementary file 1 — Supplementary Material 1: Fasting biochemical parameters at the time of ultrasonography evaluationThe table summarizes the distribution of key biochemical markers in the study cohort, including lower and higher recorded values, median, and reference ranges. Parameters assessed included fasting glycemia, triglycerides (TAGs), total cholesterol, HDL, LDL, and insulin-like growth factor-1 (IGF-1). Reference values are age-adjusted according to BC Children’s Hospital guidelines (https://www.bcchildrens.ca/endocrinology-diabetes-site/documents/igf1nml.pdf) [file 13023_2026_4332_MOESM1_ESM.pdf]

Table 1S: Fasting biochemical parameters at the time of ultrasonography evaluation

Biochemical marker	Lower Value	Higher Value	Median	Reference range	Units of measure
Glycemia	28	74	66,5	65–100	mg/dL
TAGs	42	231	67,5	20–170	mg/dL
Total cholesterol	113	223	174,5	130–200	mg/dL
HDL cholesterol	26	67	49,5	>40	mg/dL
LDL cholesterol	51	147	100,5	<130	mg/dL
IGF-1	15	225	78	According to age*	ng/mL

*TAGs: triglycerides; HDL: high density lipoprotein cholesterol; LDL: low density lipoprotein cholesterol; IGF-1: insulin-like growth factor-1. Reference values are age-adjusted according to BC Children's Hospital guidelines (https://www.bcchildrens.ca/endocrinology-diabetes-site/documents/igf1nml.pdf)
