# Supplementary material for: Muscle ultrasonography in costello syndrome: unveiling new clinical insights of a complex muscular phenotype
Source: Orphanet J Rare Dis. 2026 Apr 23;21:223. doi: 10.1186/s13023-026-04332-3 (PMC13277121; doi:10.1186/s13023-026-04332-3)
Supplement: Supplementary file 2 — Supplementary Material 2: Daily macronutrients kilocaloric intake according to reference range The table shows the distribution of participants with appropriate, below-range, or above-range daily caloric intake for carbohydrates, lipids, and proteins [file 13023_2026_4332_MOESM2_ESM.pdf]

Table 2S: Daily macronutrients kilocaloric intake according to reference range

Daily Kcal intake	Appropriate	Below range	Above range
Carbohydrates	4 (20%)	13 (65%)	3 (15%)
Lipids	17 (85%)	1 (5%)	2 (10%)
Proteins	3 (15%)	0	17 (85%)
Total	17 (85%)	0	3 (15%)

Distribution of participants (total number and % of patients) with appropriate, below-range, or above-range daily caloric intake according to LARN 2014, for carbohydrates, lipids, and proteins.
