# Supplementary figures and images for: Muscle ultrasonography in costello syndrome: unveiling new clinical insights of a complex muscular phenotype
Source: Orphanet J Rare Dis. 2026 Apr 23;21:223. doi: 10.1186/s13023-026-04332-3 (PMC13277121; doi:10.1186/s13023-026-04332-3)

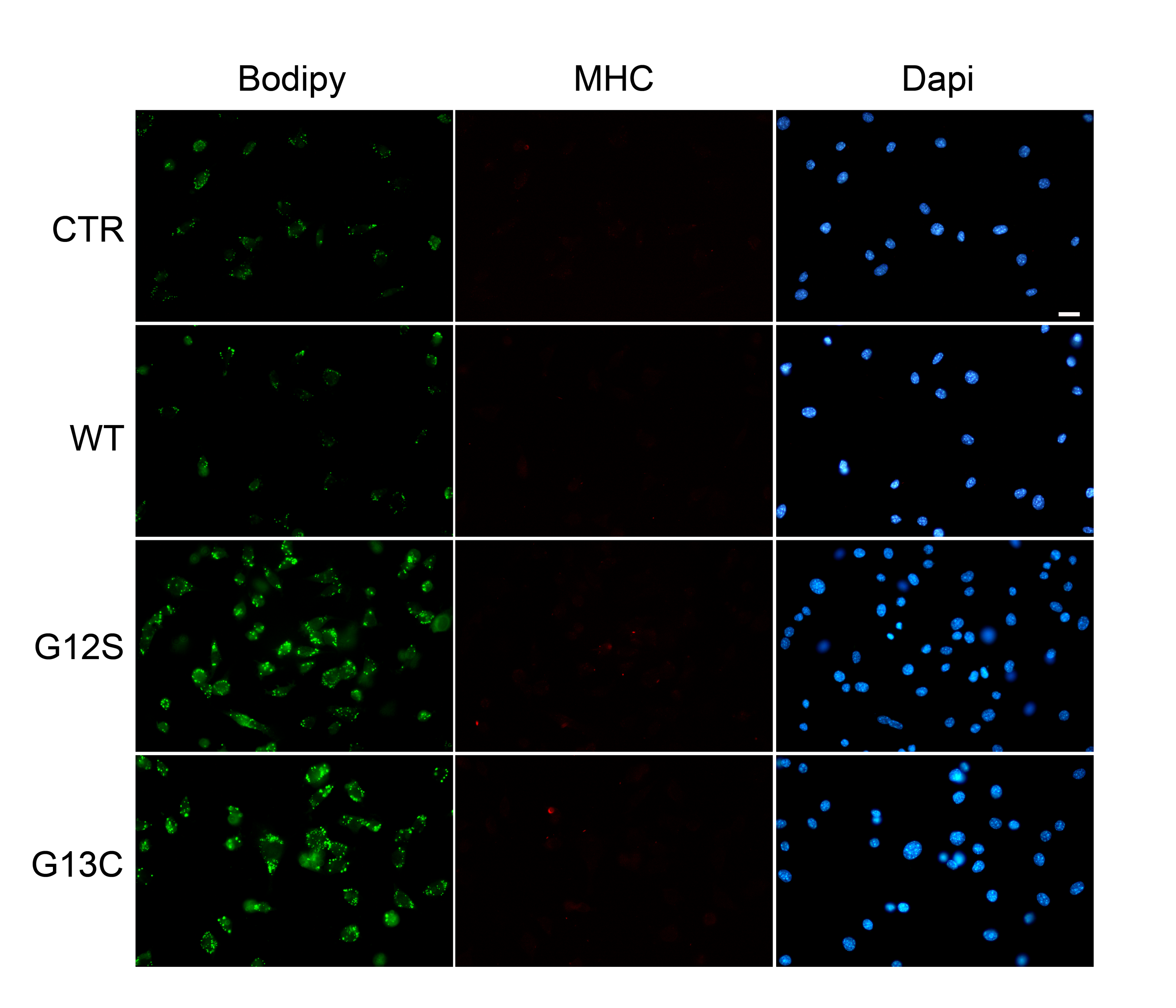

Supplement: Supplementary file 3 — Supplementary Material 3: Lipid droplets detection in proliferating muscle cells stably expressing WT and mutants HRAS Representative images showing an increased amount of lipid droplets in proliferating myoblasts expressing HRAS mutants (G12S and G13C) compared to cells expressing WT HRAS and control ones. Lipid droplets content was evaluated after fixation with 3% PFA and permeabilization with 0.5% Triton X-100 by using the fluorescent lipid-specific Bodipy dye (green). Cells were stained also with myosin heavy chain (MHC) antibody (red). Nuclei are visualized by DAPI staining (blue). Scale bar is 20 μm. [file 13023_2026_4332_MOESM4_ESM.tif]
